# Implementation of a phased medical educational approach in a developing country

**DOI:** 10.3402/gha.v8.29882

**Published:** 2015-11-11

**Authors:** Michelle R. Holm, Holly L. Burkhartzmeyer

**Affiliations:** Mayo Clinic, Rochester, MN, USA

**Keywords:** phased educational approach, sustainability, educational initiative, medical education, educating in developing countries, Haiti, healthcare

## Abstract

**Objective:**

Healthcare provider education can serve as one method for improving healthcare in developing countries. Working with providers at St Luke Hospital in Haiti, we developed a phased educational approach through partnership development, face-to-face teaching, and virtual educational tools.

**Design:**

Our novel approach included three phases: direct patient care, targeted education, and utilization of the train-the-trainer model. Our end goal was an educational system that could be utilized by the local medical staff to continually improve their medical knowledge, even after our educational project was completed. We implemented pre- and post-lecture evaluations during our teaching phase to determine whether the education provided was effective and beneficial. Additionally, we provided medical lectures on a shared file internet platform, Box.com, during the train-the-trainer phase to allow healthcare providers in Haiti to access the educational content electronically.

**Results:**

In total, 47 lectures were given to 150 medical providers, including nurses, physicians, and pharmacists. Pre- and post-lecture evaluations were administered. The mean was 30.63 (14.40) for pre-lecture evaluations and 93.36 (9.80) for post-lecture evaluations indicating improvement out of a total of 100 possible points. Our collaborative Box.com account contains 214 medical education lectures available for viewing as a constant resource to St Luke Hospital staff. Thus far, 20 of the 43 (47%) Haitian medical providers have viewed lectures, with an average of 5.6 lectures viewed per person. Qualitative data suggest that these methods improved communication between healthcare staff, promoted better ways of triaging patients, and improved job satisfaction.

**Conclusions:**

A phased educational approach can improve healthcare workers’ knowledge through partnership in a developing country. Educating local providers is one way of ensuring that in-country healthcare staff will improve their medical knowledge and expertise.

Healthcare organizations operate on six basic principles – cost, efficiency, impact, preparedness, education, and sustainability (1, 2). In an evaluation of quality of care, the impact of health education was found to be the least utilized of these; only 64% of hospitals in developing countries used education to improve outcomes (1, 2). Healthcare education in developing countries is underutilized but also an area for potential improvement.

The Mayo Clinic in Rochester, MN, partnered with St Luke Hospital in Port-au-Prince, Haiti. St Luke Hospital is an adult hospital that has an annual census of 150,000 patient visits and includes an emergency department, intensive care unit, and cholera rehydration center. The partnership began after the 2010 Haiti earthquake (3), through responding to disaster relief patient care needs requested by St Luke Hospital. These initial efforts generated a strong relationship which is built upon a foundation of trust between the two institutions.

There is little relevant research into long-term, partnered, educational methods used in developing countries. However, roughly 6,000 Americans participate in medical mission trips each year (1). Many participants could potentially benefit from informed guidance before beginning undertaking these trips. We hypothesized that through development of a novel phased educational approach, including virtual educational methods, we could improve the medical knowledge base of the healthcare staff at St Luke Hospital, and engage them in initiating their own continuing education.

## Methods

The phased educational approach was developed by our institution and included three phases: direct patient care, educational training, and utilization of the train-the-trainer model. The study population for each of the phases included 150 healthcare professionals employed at St Luke Hospital, including physicians, nurses, and pharmacists.

### Phase 1

Phase 1, direct patient care, involved working side by side with our Haitian healthcare colleagues to assist them in taking care of their patients and to build trust between the two organizations. Medical staff from our institution traveled to Haiti every other week for the first 6 months to help provide care to patients with trauma related to automobile crashes, injuries sustained from the earthquake in 2010, stroke, hypertension, and cholera, among other conditions.

During these visits, we performed a continuous needs assessment to determine which areas of education our colleagues deemed most valuable to them (1). The benefits of an educational needs assessment include acquiring the necessary knowledge to properly plan activities, determining the best logistical approach for providing and using the resources available, and finally, deciding which educational avenues will be most engaging to meet the expectations and needs of the audience. Our needs assessment verified that evidence-based educational approaches were an appropriate resource for reinforcing best practice guidelines and evidence-based medicine to ultimately improve patient health and outcomes (4, 5). We collected and analyzed needs assessment data following each team's recommendations. We identified relevant performance gaps (5), after which we measured skills to assess our colleagues’ areas of strength and areas of need.

**Fig. 1:**
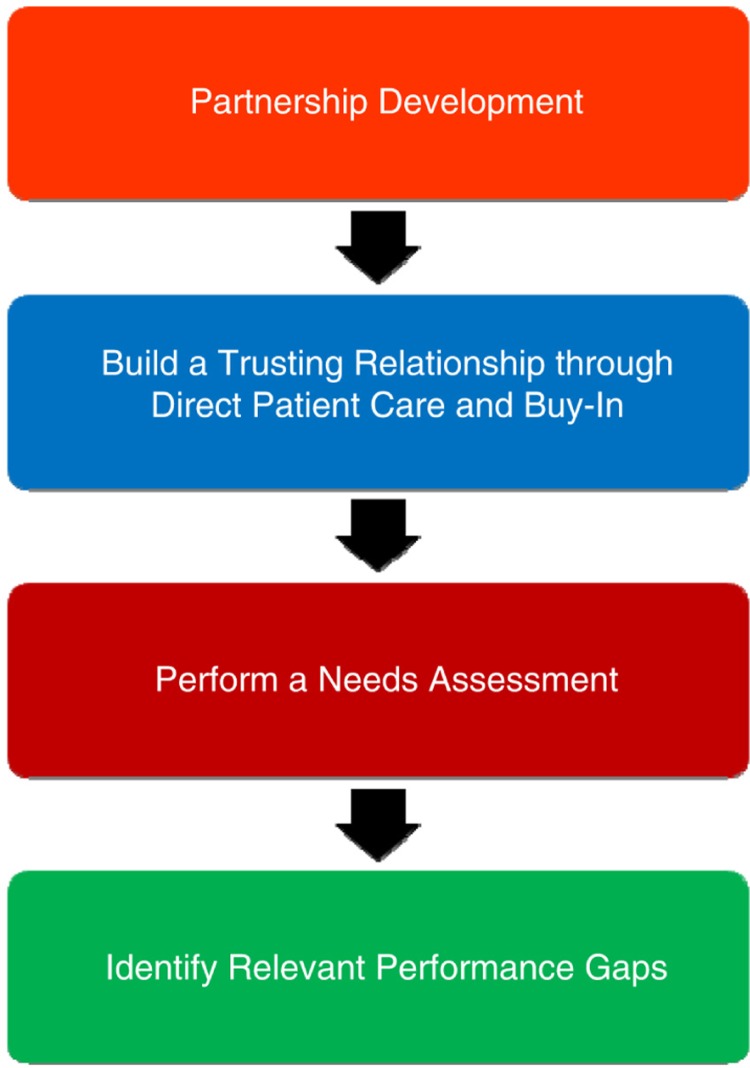
Phase 1 – building a relationship and performing a needs assessment with our partner, St Luke Hospital, through direct patient care.

Phase 1 provided the opportunity to establish the aims and to formulate the vision, mission, and goals of our partnership (6). Our colleagues’ input and buy-in (acceptance of and willingness to actively support and participate) were considered essential steps in the process by which to ensure that each staff member agreed with the educational plan (7). The stepwise approach used within Phase 1 is shown in Fig. 1.

Prioritizing the list of educational topics involved a collaborative logistical process (e.g. best time of day to provide education so that each staff member could attend, timing of the teaching around patient workload, and composing groups small enough for question and answer sessions to be most useful). Involving key stakeholders such as St Luke Hospital leadership as well as the direct patient care staff at the hospital built collaboration prior to the implementation phase (8). Additionally, we collaborated with Operation Blessing International, a nongovernmental organization, which served as our logistics and accommodations partner in Haiti.

### Phase 2

During the second phase of our partnership, we initiated in-person educational sessions. These sessions included evidence-based approaches such as targeted lectures, clinical vignettes, and multimodal techniques to promote knowledge acquisition provided to various specialties within St Luke Hospital (3). We established performance-based objectives, developed performance-assessment measures, and set the scope of activity and expected outcomes (5).

It was important to fully understand our Haitian colleagues’ needs to ensure that the most suitable approach was chosen for each educational session. We recognized that delivering education that diverges too far from frequently encountered clinical topics tends to be defective and deteriorate from the start. Therefore, we listened carefully to their needs and sought their buy-in before beginning the implementation of the educational activities (9). Examples of each type of educational activity are listed in Table 1.

**Table 1:** Phase 2 – examples of each type of educational activity presented to our study population consisting of physicians, pharmacists, and nurses

Evidence-based educational approaches	Physicians	Pharmacists	Nurses
Targeted education	Hypertension Stroke Infectious disease Diabetic ketoacidosis	Toxicology Medication calculations IV compatibility	Asthma Glucose control Dysphagia
Clinical vignettes	Sepsis Acute kidney failure Complex intensive care Pain management	Drug–drug interactions Adverse effects Drip rates	Signs/symptoms of asthma vs. COPD Triage of patients with sepsis
Multimodal teaching	Advanced cardiac life support Intubation Bedside sonography Burn treatment	Pharmacy computerized inventory program (3) Substitution of equivalent medications	Pressure ulcer prophylaxis Hand hygiene Cardiac monitoring Inhaler education

COPD, chronic obstructive pulmonary disease; IV, intravenous.

When presenting educational topics, we focused on patient-case-based presentations, rather than presenting in the traditional didactic lecture fashion. Our staff kept PowerPoint slides brief in order to engage audience participation and discussion. Translation from English to Haitian Creole was effective using simple, straightforward slides (10).

After first presenting background information on the medical topic, we moved on to various case examples; described when audience members might encounter the need for the specific, targeted, education presented; and explained how to apply the education to currently hospitalized patients. The staff from our institution kept the audience involved by asking questions, and encouraging participation by allowing audience members to demonstrate how they would treat a patient using the medical education they just received. After each lecture, signed certificates were given to the participants, as a way of verifying that they engaged in and completed the educational activity. Participants kept their certificates for later use and as a record of the educational lectures they had completed.

Pre- and post-lecture evaluations were conducted to verify that the education was relevant to the participants’ practice and educational needs and that the method of delivery was beneficial (4). Staff members from our institution administered four to six questions both before and after the various educational sessions given to our study population. We administered these evaluations to verify that the educational topics chosen provided opportunities for growth in the knowledge of what was previously unfamiliar material. Post-lecture evaluations were sought to measure whether the objectives of the training were met after each training session.

### Phase 3

The train-the-trainer method was used in Phase 3. This supported our Haitian colleagues in teaching each other to create an environment of continuous education within the hospital, regardless of whether outside institutions, such as ours, were present. This is a technique that calls for action-oriented learning (4). Our institution shifted from the role of the primary deliverer of education to a resource, as our colleagues began to provide educational topics to one another. Phase 3 required both parties to share an equal role in creating a sustainable environment; and accountability was at the forefront of our initiative.

The Plan-Do-Study-Act (PDSA) model was chosen as our educational technique in the train-the-trainer approach. Our institutions planned our approach carefully, practiced our educational method, and studied the results to determine which methods worked well. We made adjustments accordingly; and implemented the adjustments during the testing of the train-the-trainer education.

We found that e-learning could be a valuable tool to provide updated medical information to healthcare colleagues in developing countries, enabling them to engage in continuous learning when there are limited teaching resources (11, 12). Lectures previously given were assembled on a secure file share internet platform, Box.com, as a means to review prior lectures at any given time and to continuously engage in virtual medical education. We organized the lectures by language to assure that our colleagues could easily sort by their preferred language.

Box.com views were tracked to verify usage and to help determine which past lectures were most valuable to our Haitian colleagues. Our Box.com account includes 43 collaborators, the majority being Haitian medical providers. Several staff members in each specialty were identified as primary educators to initiate the transition to Phase 3. Staff from St Luke Hospital could access the education from any computer connected to the internet and choose from hundreds of lectures to have ongoing education for themselves and their peers.

Our institutional review board reviewed this study in accordance with the US Code of Federal Regulations, 45 CFR 46.

## Results

Phase 1 consisted of performing a needs assessment and identifying performance gaps after having built a trusting partnership. Relevant performance gaps were documented and discussed between our institutions to ensure that the educational lectures, which would be completed during Phase 2, met the needs of the assessment. Bedside care skills were taught. They included how to diagnose and treat infectious diseases, assess trauma victims, and triage patients on the basis of acuity.

During Phase 2, a total of 47 lectures were delivered to 150 staff members including nurses, physicians, and pharmacists. Each lecture had its own pre- and post-lecture evaluations based on the content of that specific lecture. The mean (SD) was calculated for the pre-lecture and post-lecture evaluations to determine the consistency of the evaluation results among the participants and their knowledge gain after receiving the education. Table 2 displays the SD for pre-lecture evaluations that were calculated at 30.63 (14.40) out of a possible 100 points. Post-lecture evaluations demonstrated a mean (SD) of 93.36 (9.80) out of a possible 100 points for the study population shown in Table 2.

**Table 2:** Mean (SD) findings associated with pre-lecture and post-lecture evaluations given to our study population during Phase 2

Findings	Pre-lecture assessment	Post-lecture assessment
Mean (out of a total possible point value of 100)	30.63	93.36
SD	14.40	9.80

**Fig. 2:**
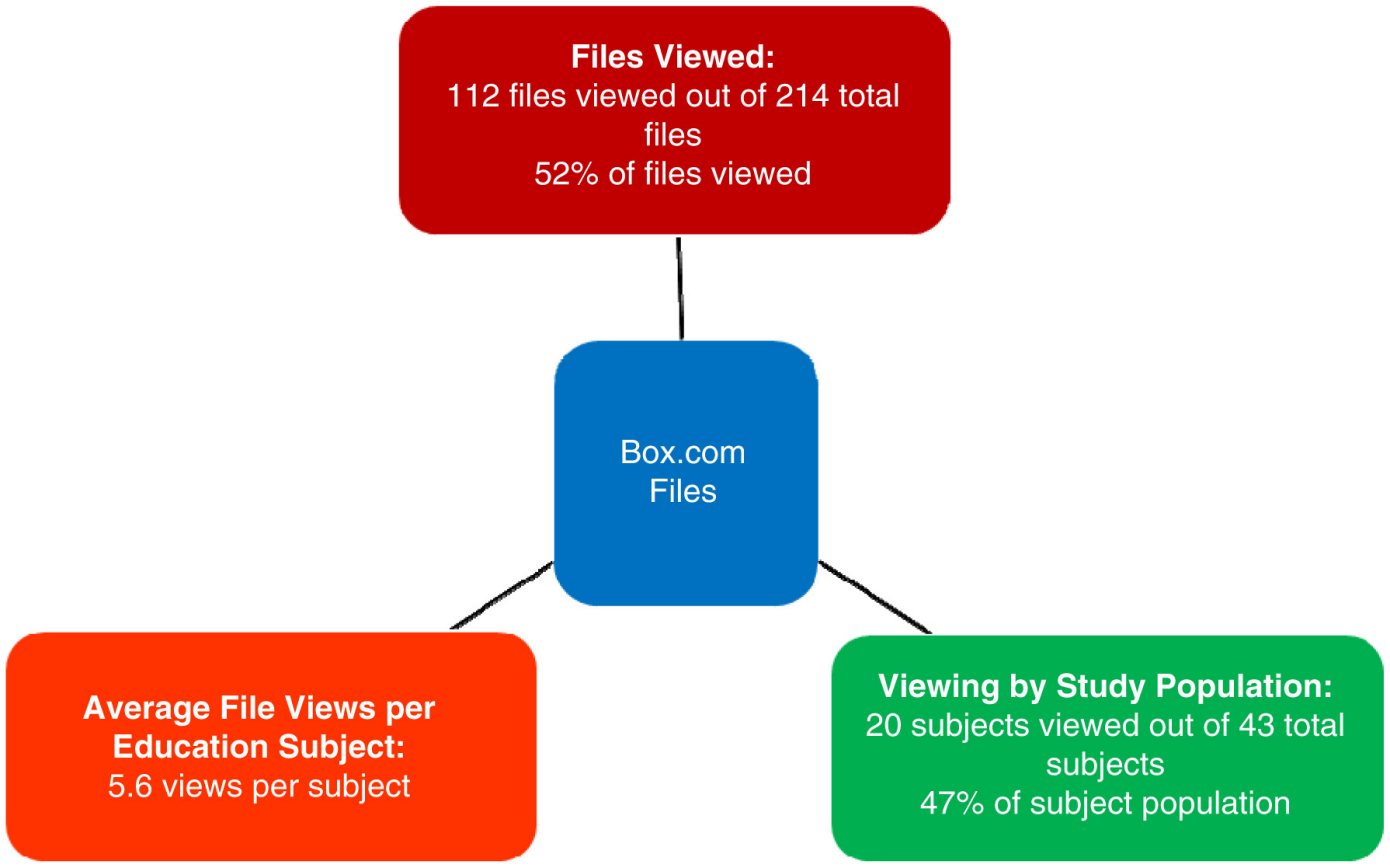
Phase 3 – medical educational file viewings in relation to viewing by subjects within the study population, supplemented by average files viewed per subject.

Phase 3 consisted of uploading 214 medical education files, including lectures, guidelines, and evaluations, to Box.com. Thus far, 112 of the files have been viewed and 20 of the 43 (47%) Haitian medical providers have viewed the lectures. An average of 5.6 lectures was viewed per medical provider viewer (Fig. 2). Of note, the most highly viewed lectures included cardiology and stroke topics for physicians; asthma, chronic obstructive pulmonary disease and blood sugar education for the nursing staff; and drug–drug interactions and dosing calculations for the pharmacists.

Qualitative data from our approach highlight some of the improvements observed following the educational sessions. St Luke administrative staff members stated that their nurses and physicians were better able to prioritize patients in need of emergent care following the triage education provided. Improved medical chart documentation was also observed following the educational lectures provided. Ultrasound education provided to the physicians has resulted in the newfound knowledge of being able to calculate a patient's ejection fraction (EF) thereby improving the likelihood of a patient who can be aided by medical care and be seen more rapidly by the healthcare team.

Nursing education improved the confidence level of the nursing staff. This was witnessed through nurses approaching the physicians more readily, rather than waiting for a prolonged period of time, during which they may have medical concerns regarding their patients. An example of immediate adjustment to practice involves observation of nursing staff, either pushing intravenous medications or running intravenous drips wide open, prior to receiving an educational lecture on drip rate calculations. Immediately following the lecture, we witnessed the nurses calculating the drips-per-minute rate using the calculators on their phones before determining how quickly they needed to run the fluid intravenously.

St Luke pharmacists were encouraged by our staff to present medical lectures to nurses along with the initiation of collaborating with physicians and nurses during multidisciplinary rounds. This improved the level of trust between the staff. Pharmacists have also vocalized improved job satisfaction attributed to the increased number of medication-related questions asked by colleagues following the educational presentations.

## Discussion

Before initiating our partnership with St Luke Hospital, our medical teams reviewed several leadership principles, including listening, empathy, awareness, persuasion, and conceptualization, as well as Haitian cultural considerations. The purpose was to ensure that we were culturally aware and knowledgeable about entering a partnership with another hospital (7, 13, 14). We focused on Global Health Training Guidelines such as developing a well-structured program; considering local needs and priorities; maintaining long-term partnerships; clarifying goals, expectations, and responsibilities through agreements; and developing, implementing, and improving regular training. All of these should occur at the same time as encouraging non-threatening communication (15, 16). We also relied heavily on the buy-in of our colleagues. We recognized that profound knowledge usually comes from outside the system and is useful only if invited in and received by participants with some eagerness to learn and improve (4).

By the end of Phase 1, we had built a solid, trusting, relationship with our colleagues at St Luke Hospital and had completed a thorough needs assessment. During Phase 2, we used proven educational methods and evaluation tools to deliver educational lectures requested by St Luke Hospital staff to determine whether there had been educational improvement in the subject matter being taught.

The educational lecture results depict a high mean percentage for the post-evaluation compared to the pre-evaluation. There was low variability in the post-evaluation compared with the pre-evaluation, and participants consistently improved their scores from the pre-evaluation to the post-evaluation. The evaluations were conducted by our institution to assess gaps in the participants’ medical knowledge. They also provided a resource to staff and served as a basis for adjusting future presentations in line with the feedback. Scores improved from pre-evaluation to post-evaluation. Staff vocalized their understanding of the material presented and demonstrated this understanding through patient care activities following the educational activity.

Subjectively speaking, we found medical knowledge improved after educational lectures. This was evident by observing patient care activities before and after educational lectures were delivered. For example, ultrasound education provided a means to better triage patients based on their EF score, nursing education solidified drip rate calculations, and pharmacists were integrated into the healthcare team through multidisciplinary rounding.

The transition into Phase 3 was composed of providing lectures and guidelines on Box.com. Reviewing lectures and guidelines on Box.com was used as an educational tool to facilitate staff revising lectures with the newest available guidelines and/or teaching lectures to each other. Encouraging our colleagues to begin teaching themselves and each other was imperative to improving their long-term medical knowledge. Although this could not be assessed in this short period of time, we expect that delivering continuing education will contribute toward sustainable long-term goals (17).

Given that many of the staff were unaccustomed to computers, Box.com navigation and using the internet (*versus* textbooks) for medical educational purposes was a challenge for many. However, a 47% ‘view rate’ within the first 6 months was a positive finding. Usage data (views and downloads) were utilized to verify distance-based education and this was deemed valuable by St Luke Hospital staff. Of note, staff reported an increased comfort level with topics after having reviewed them again on Box.com, following the initial teaching. The requested educational lectures continue to be used by colleagues in Haiti months after staff members at our institution originally gave the lectures. They continue to serve as an educational resource to St Luke Hospital staff as part of the train-the-trainer method.

Our phased educational approach provided the structure necessary to develop a solid platform for the utilization of education to foster improved patient outcomes. Measuring improved patient outcomes and associating improvement with any one factor, such as education, is a challenge. However, qualitative data showed the benefits of improved documentation within medical charts, initiation of multidisciplinary rounds, and improved communication between medical staff. All of these suggest that our educational partnership has been beneficial in the eyes of our institution and the staff at St Luke Hospital.

Although our phased educational approach is novel, we found reinforcement of our findings in shared experiences of related initiatives through a recent literature search of databases: PubMed and CINAHL. The importance of buy-in was emphasized in a Haitian nursing education initiative. Clark et al. (18) found that a continuing education initiative in a low-resource setting is possible when there is commitment and engagement for nursing continuing education at all levels of the organization. Nadas et al. (19) looked at the continuing professional development needs of Haitian physicians and found that continuing medical education activities were key areas of need, in terms of supporting the clinical and professional work of physicians.

### Challenges

We learned about the benefits and pitfalls of pre-and post-lecture evaluations. Benefits included ease of administration of the tests, low cost of administration, and straightforward scoring – all very useful in the context of developing countries (4). The challenges of pre- and post-lecture evaluations included keeping the information confidential and promoting an environment of trust to ensure that audience members feel comfortable providing their answers *versus* looking at someone else's answers or avoiding the evaluation altogether. We learned that performance-based recognition, such as public recognition or certificates of achievement, promoted improvements, and the desire to learn in a positive environment (4).

Our institution found that time and buy-in were our main challenges. It is more time-intensive to teach education principles and explain how to teach, than it is to simply give a lecture. Teaching PDSA and learning how to deliver effective educational offerings, takes considerable time and effort. Therefore, a long-term commitment is necessary to ensure successful outcomes. PDSA also introduced a different method of learning considering our Haitian colleagues stated that their educational background consisted of the memorization of facts *versus* practicing, doing, studying, and acting. Introducing a change in the way education is delivered was a major challenge.

However, the primary challenge for St Luke Hospital staff has been time constraints due to a high patient-to-provider ratio. Staff members have communicated that they do not have enough time away from patient care to create lectures, practice, and provide the content to colleagues. However, as a starting point, access to reviews of previous lectures through Box.com, was deemed beneficial by the Haitian medical staff.

### Limitations

Very few articles have been published about providing long-term education systems in an underserved, resource-constrained, setting – leading us to create our own educational approach. Further resources would have been beneficial in allowing us to adopt more efficient ways of educating in a developing country and to overcome barriers encountered when introducing and managing change in institutions such as hospitals.

### Looking ahead

Train-the-trainer provides the means for St Luke staff to begin providing education to one another as well as engaging in continuing education. This is also expected to become a future requirement for Haitian healthcare workers. Continuous education is a professional lifelong learning process, and whether a requirement or not, is better for the providers and also their patients. By starting on a small scale, we hope to improve upon this process and expand to other institutions in need of medical education in the developing world.

## Conclusions

Our phased educational approach provides one way of improving the knowledge of healthcare workers in an underserved setting (3–5, 20). The approach is replicable and can potentially be used by other individuals and organizations partnering with medical institutions in developing countries. Further research is required to determine how these interventions will impact patient outcomes and whether distance-based education will become a sustainable initiative in future years.
